# NRF2 signaling drives chemoresistance in NSCLC and glioblastoma

**DOI:** 10.1007/s11010-026-05747-z

**Published:** 2026-09-19

**Authors:** Izadora de Souza, Linda Karolynne Seregni Monteiro, Ana Beatriz da Silva Teixeira, Camila Banca Guedes, Karoline Almeida Lima, Cláudio Henrique Dahne de Souza Filho, Izabela Amélia Marques de Andrade, Maria Carolina Clares Ramalho, Beatriz Silva de Oliveira, Mariana Lazarini, Clarissa Ribeiro Reily Rocha

**Affiliations:** Department of Clinical and Experimental Oncology, Federal University of São Paulo (UNIFESP), São Paulo, 04037-003 Brazil

**Keywords:** NRF2, Temozolomide, Cisplatin, Glioma, Lung cancer, Ferroptosis

## Abstract

Although great advances have been made in cancer treatment, lung cancer and glioblastoma patients continue to present a dismal prognosis, mainly due to drug resistance. Evidence suggests that NRF2 and glutathione (GSH) play a fundamental role in chemotherapy resistance. Notably, GSH depletion by buthionine sulfoximine (BSO) has been demonstrated to sensitize human tumor cells to a wide variety of chemotherapeutic agents. However, the incorporation of BSO into standard chemotherapy regimens lacks a robust clinical rationale. In this study, we aimed to investigate the mechanisms governing differential sensitivity to chemotherapy in lung cancer and glioblastoma cells to identify novel strategies to enhance outcomes in these malignancies. For this purpose, we analyzed several cellular responses in cell lines exhibiting distinct sensitivities to temozolomide (TMZ) and cisplatin. Our findings indicate that the NRF2/GSH pathway plays a crucial role in TMZ and cisplatin resistance and that pharmacological GSH depletion enhances the sensitivity of NRF2-high, chemotherapy-resistant cells to these agents. Furthermore, BSO in combination with TMZ or cisplatin enhanced chemotherapy-induced cytotoxicity and engaged both apoptotic and ferroptosis-related responses in NSCLC cells. These findings provide preclinical evidence supporting further investigation of NRF2/GSH targeting as a strategy to overcome chemotherapy resistance.

**Graphical abstract:**

**Figure:**
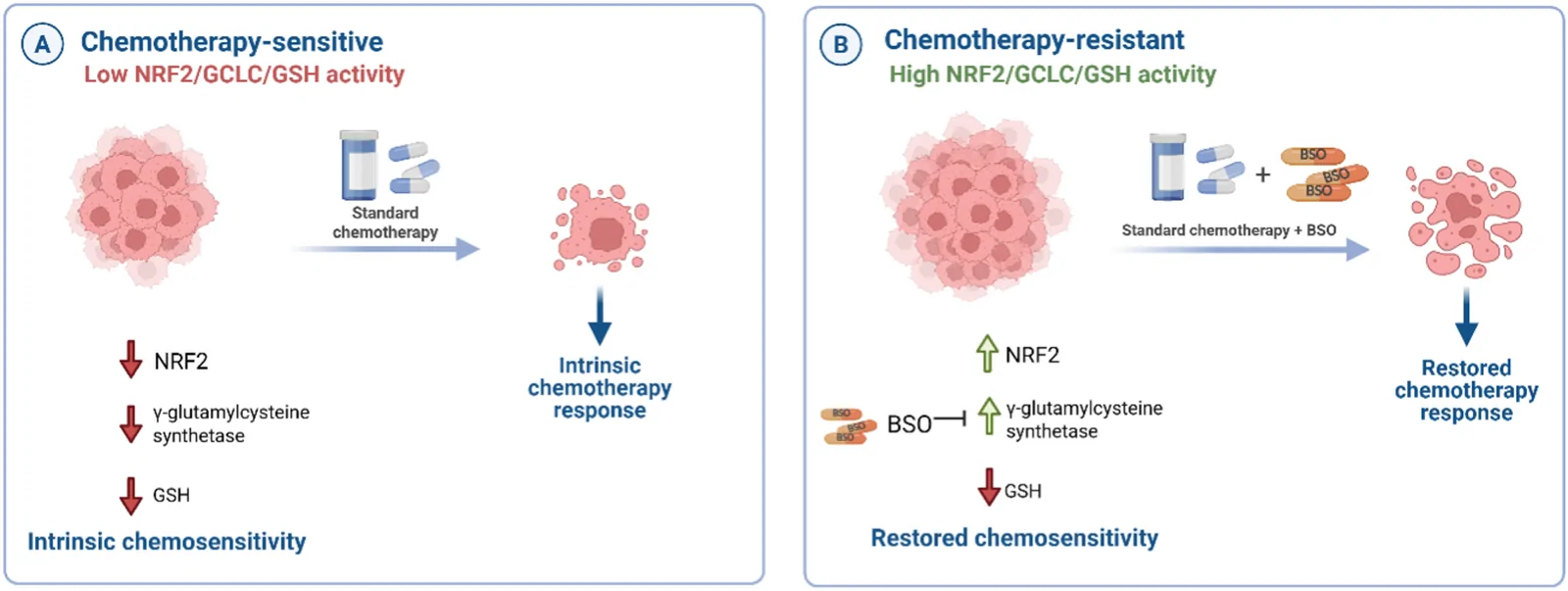

**Supplementary Information:**

The online version contains supplementary material available at https://doi.org/10.1007/s11010-026-05747-z.

## Introduction

Cancer remains a major global health challenge and a leading cause of mortality worldwide. Despite significant advances in diagnosis and treatment, an estimated 2,041,910 new cancer cases and 618,120 cancer-related deaths are projected to occur in the United States in 2025 alone, underscoring the continued need for more effective therapeutic strategies [1]. Among these, lung cancer emerged as the leading cause of cancer mortality, particularly among individuals aged 50 years and older [2]. Non-small cell lung carcinoma (NSCLC) subgroup is responsible for about 85% of cases of lung cancer [3, 4]– [5], and typically displays significant resistance to cisplatin (first line treatment) [6, 7]. While advances in early detection and therapeutic interventions have markedly improved survival rates in NSCLC, the emergence of chemoresistance remains a critical barrier to durable treatment responses, frequently leading to disease relapse and reduced long-term clinical benefit [1, 8].

Similar challenges are observed in malignant brain tumors, which, despite their relatively low incidence, represent the leading cause of cancer-related mortality among children and young adults under 20 years of age [2]. Of notice, glioblastoma is the most aggressive and frequent type of malignant brain tumors [9, 10]. Temozolomide (TMZ) is the first-line therapy for glioblastoma, and it has improved survival rates by about two months [11, 12]. Nevertheless, patient prognosis remains dismal, with a median survival of approximately 15 months and a 5-year overall survival rate of ~ 7%, largely driven by therapy resistance [13, 14].

Significant studies have greatly expanded our knowledge on tumor biology, leading to improvement of cancer treatment; however, chemotherapy resistance remains a huge clinical obstacle to the patient’s cure [15]. Thus, approximately half of the patients treated with cisplatin commonly exhibit primary resistance or progressively develop resistance to multiple therapeutic agents [7, 16], and the efficacy of TMZ diminishes significantly in recurrent cases [17]. Importantly, while many molecular pathways were described to be involved in drug resistance, it is well-established that one of the pivotal pathways contributing to resistance against cisplatin and TMZ is the antioxidant pathway [18, 19].

Nuclear factor erythroid 2-related factor 2 (NRF2) functions as a central regulator of the cellular antioxidant program, playing a key role in the maintenance of redox balance [20]. Of note, NRF2 governs the expression of key enzymes required for glutathione (GSH) biosynthesis, including glutamate–cysteine ligase modifier subunit (GCLM) and glutamate–cysteine ligase catalytic subunit (GCLC), as well as enzymes involved in GSH recycling and utilization, such as glutathione reductase (GR) and glutathione S-transferases (GSTs) [21]. As a highly abundant low-molecular-weight thiol, GSH is essential for preserving intracellular redox homeostasis through its capacity to scavenge reactive species [22]. In the context of chemotherapy, GSH can directly conjugate cisplatin via its reactive thiol group, thereby limiting DNA adduct formation and reducing drug efficacy [23]. Also, it can control the reactive oxygen species triggered by TMZ treatment [24]. Indeed, high NRF2 and GSH levels have been related to increased resistance to various chemotherapeutic drugs in different cancers, including lung and brain tumors [25–28]– [29]. GSH downregulation induced by pharmacological inhibition or NRF2 silencing intensified the cytotoxicity of TMZ and cisplatin in glioma and NSCLC cells, respectively, enhancing susceptibility to DNA damage and cell death [23, 25, 26].

Consequently, the combinatorial therapy of GSH inhibition with anticancer drugs has emerged as a novel therapeutic strategy for numerous human cancers [23, 30–32]– [33]. L-buthionine [S, R]-sulfoximine (BSO), a potent GSH inhibitor, has been widely utilized to deplete cellular GSH [34]. Studies in vitro and in vivo have supported the potential of BSO as an adjuvant to conventional chemotherapy, yielding promising results [25, 35, 36]– [37]. BSO treatment may trigger different types of cell death, such as apoptosis and ferroptosis, but the molecular pathways involved in the combination of BSO with chemotherapy remain unclear [31, 38, 39]– [40].

In this study, we investigated the molecular mechanisms underlying resistance to temozolomide, cisplatin, and BSO in lung carcinoma and glioma cells. Our findings confirm the pivotal role of NRF2 in chemotherapy and BSO resistance in cancer. Resistant tumor cells exhibited elevated NRF2 levels, and NRF2 silencing significantly increased their drug sensitivity. BSO treatment demonstrated a substantial impact on tumors with low GCLC levels, which are characterized by shorter overall survival compared with higher GCLC levels in glioma patients. Furthermore, GSH depletion significantly sensitized NRF2-upregulated cells to chemotherapy by inducing apoptosis and ferroptosis in NSCLC. Our results suggest a novel, powerful approach to improve chemotherapy efficacy for patients who do not benefit sufficiently from chemotherapy alone due to apoptosis-acquired resistance.

## Materials and methods

### Cell lines and culture conditions

Certified human glioma cell lines U251MG and T98G and human lung carcinoma cell lines A549 and NCI-H23 were kindly provided by Prof. Bernd Kaina (Germany) and by Prof. Mari Sogayar (University of São Paulo, Brazil), respectively. U251MG, T98G and A549 cells were grown in DMEM medium (Invitrogen, Life Technologies, Carlsbad, CA, USA), while the NCI-H23 cell line was cultured in RPMI medium (Invitrogen). Both culture media were supplemented with 10% FCS (fetal calf serum; Cultilab, Campinas, SP, Brazil) and 1% antibiotic-antimycotic. Cells were grown at 37° C in a humidified 5% CO_2_ atmosphere. All cell lines were authenticated by STR profiling and tested negative for mycoplasma contamination.

### Chemicals and reagents

Temozolomide and ferrostatin-1 were purchased from Cayman Chemical Company (Michigan, USA) and were dissolved in DMSO under sterile conditions to a concentration of 50 mM and 1 mM, respectively and stored at −80^0^ C. Cisplatin (Sigma-Aldrich, Taufkirchen, Germany) was diluted in PBS 10x (Phosphate-buffered Saline) in a stock solution of 1 mM and maintained at −80^0^ C. L-Buthionine-sulfoximine (BSO) was freshly prepared in PBS 1x at different concentrations. Z-Vad-fmk (Promega, Madison, WI, USA) was provided at 20 mM in DMSO and stored at −20^0^ C.

### Cell survival analysis

Clonogenic assays were performed by initially seeding 2 × 10^3^ cells/well in 6 well-plates. After 24 h, the cells were treated with the compound of interest and maintained at 37 °C and 5% CO_2_ for 8 to 10 days, then the colonies were fixed with 4% formaldehyde for 15 min, stained with 1% crystal violet (Sigma–Aldrich) and photographed. The colonies with more than 30 cells were manually counted for quantitative analysis. For cell viability assays using XTT, a total of 1 × 10^4^ cells were seeded in a 12 multi-well plate and incubated with different drugs for 72–120 h. In some experiments the cells underwent a pre-treatment phase for 24 h with BSO. Different BSO concentrations were used depending on the experimental endpoint and assay conditions. Higher concentrations (1–5 mM) were employed in short-term XTT viability assays to evaluate acute metabolic effects, whereas lower concentrations (30 µM) were used in clonogenic assays, which assess long-term proliferative capacity under low-density conditions and are inherently more sensitive to sustained oxidative stress. After that, the cells were washed with PBS 1x and incubated with XTT reagent kit, following the guidelines of the manufacturer (Roche, Basel, Switzerland). The percentage of cell survival was determined by calculating the ratio of absorbance values obtained from the experimental samples to those of the untreated control cells, which served as the reference point in the analysis.

### Flow cytometry (γH2AX, and active caspase-3 analysis)

A total of 1.2 × 10^5^ cells/well in a 12-well plate and treated with the specified drugs 24–48 h. Then, cells were collected and fixed with 1% formaldehyde and 70% ethanol. The ethanol-fixed cells underwent a series of steps, including blocking, permeabilization, and overnight incubation at 4° C with a primary mouse monoclonal antibody targeting γH2AX (Ser-139) (Upstate Biotechnology, Lake Placid, NY, USA) at a 1:500 dilution, or mouse anti-active caspase 3 (BD, Pharmigen, San Diego, CA, USA) at a 1:50 dilution for 2 h at room temperature. Samples stained with γH2AX were then exposed to an anti-mouse FITC secondary antibody (Sigma-Aldrich) at a 1:200 dilution for 2 h at room temperature. Subsequent analysis was conducted using a BD FACSCalibur flow cytometer (BD Biosciences), and the percentage of γH2AX-positive cells and caspase-3 analysis were calculated by using the FlowJo software (BD Biosciences).

### Lipid peroxidation analysis

1 × 10^5^ cells/well were seeded in 12-wells plates and exposed to the specified compounds for 24–48 h. Subsequently, the cells were harvested, washed with PBS 1x, and re-suspended in a solution containing 2 µM BODIPY C11 (581/591) probe (Invitrogen) diluted in 1x PBS. Following a 30-minute incubation period, the samples were analyzed using a BD FACSCalibur flow cytometer. Data analysis was conducted using the FlowJo software (BD Biosciences).

### Glutathione quantification

1 × 10^4^ cells/well were plated in 96 multi-well plates. After 24 h of treatment, reduced glutathione levels were quantified using the GSH-Glo Assay (Promega, Madison, WI, USA), following the manufacturer’s instructions. Luminescence was measured using a Glomax-Multi Detection System Luminometer (Promega). A standard curve was obtained by serial dilutions of a GSH standard solution to calculate the concentration of GSH in the samples.

### Western blot

The detection of endogenous proteins level was performed as previously described [41]. The following antibodies were used: anti-NRF2 (1:500) (#ab137550 Abcam, Cambridge, UK), anti-GCLC (1:1000) (#ab207777 Abcam), anti-GCLM (1:1000) (#ab126704 Abcam), and anti-βactin (1:1000) (#4967 Cell Signaling).

### Database analysis

RNA-seq expression data, clinical and molecular information of glioma patient samples were obtained from the GlioVis data portal for visualization and analysis of brain tumor expression datasets (http://gliovis.bioinfo.cnio.es/). For GCLC expression, and survival analysis, results were extracted from the CGGA (*n* = 633), TCGA (*n* = 663), and REMBRANDT (*n* = 397) cohort. Analysis of the differences in overall survival between two groups (GCLC Low and GCLC High) was performed using Kaplan-Meier curves and the log-rank test. For TCGA, CGGA and REMBRANDT the median of GCLC expression was used as the cutoff point, and correspond to 10.44, 3.95, and 9.19, respectively. OS was defined as the time between the date of sampling and the date of death (for deceased patients).

### Statistical analysis

The statistical analyses were performed with the GraphPad Prism 8 software (GraphPad Software Inc., San Diego, CA, USA). All results were represented as the mean ± SEM or SD of at least two or three independent experiments, each performed in duplicate. Two-way ANOVA and unpaired two-tailed Student’s t-test were used to evidence the significant difference among data sets, followed by Tukey’s Multiple comparison post-test (ns = not statistically significant, *p < 0.05, **p < 0.01, ***p < 0.001, ****p < 0.0001).

## Results

### Differential response to chemotherapy in glioblastoma and NSCLC cells

**Fig. 1:**
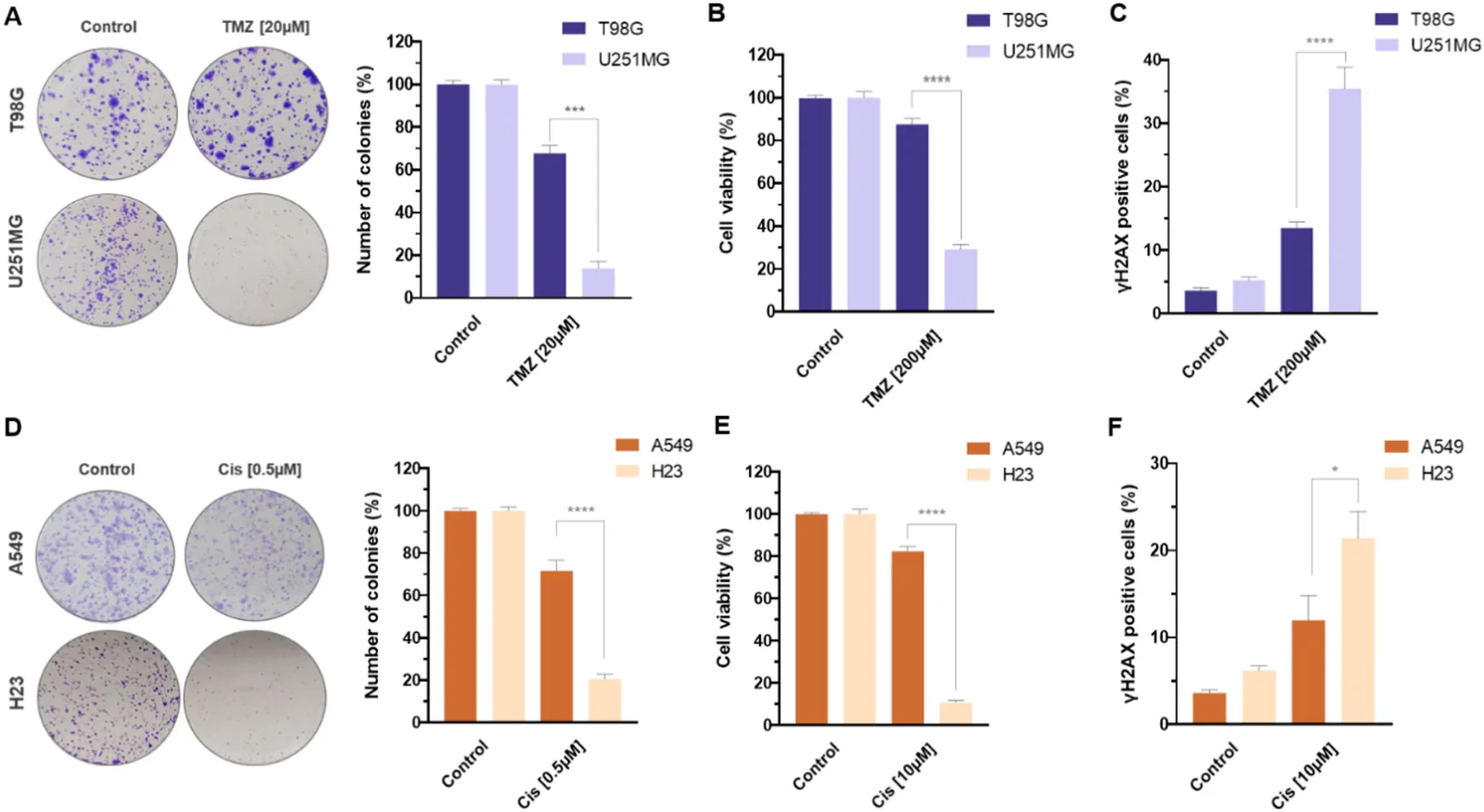
Cellular viability upon chemotherapy treatment in glioblastoma and NSCLC cells. **A** Representative image and quantification of total colonies formed after TMZ treatment (20 µM). **B** Cell viability was analyzed after treatment with TMZ (200 µM) for 120 h and measured by XTT assay. **C** Flow cytometry analysis of the percentage of γH2AX-positive cells after treatment with TMZ (200 µM) for 48 h. **D** Representative image and quantification of total colonies formed after cisplatin treatment (0.5 µM) in comparison to controls. **E** Cell viability was analyzed after treatment with cisplatin (10 µM) for 72 h measured by XTT assay. **F** Flow cytometry analysis of the percentage of γH2AX-positive cells after treatment with cisplatin (10 µM) for 24 h. Values are mean ± SEM of three independent experiments, ns = not statistically significant, *P < 0.05, **P < 0.01, ***P < 0.001, ****P < 0.0001

Clonogenic assays were initially performed to analyze the pattern of sensitivity to chemotherapy in glioblastoma and NSCLC. U251MG and NCI-H23 cells showed higher sensitivity to TMZ and cisplatin in comparison with T98G and A549 cells, respectively (Fig. 1A and D). Similarly, both cell lines presented a significant reduction in viability, with only 20% of viable cells after TMZ treatment in U251MG and 10% after cisplatin treatment in NCI-H23, whereas no significant reduction in the viability of T98G and A549 cells was observed (Fig. 1B and E). We also measured the γH2AX-positive cells after treatments since phosphorylation of the histone H2AX (γH2AX) is a widely used marker for genotoxic stress. Again, U251MG and NCI-H23 showed a higher sensitivity and a significant increase in γH2AX staining after 48 h and 24 h of TMZ and cisplatin treatment in comparison to T98G and A549, respectively (Fig. 1C and F). Altogether, these data demonstrated that T98G and A549 are significantly more resistant to chemotherapy when compared to U251MG and NCI-H23.

### Chemotherapy-sensitive cells display lower levels of GSH and NRF2

**Fig. 2:**
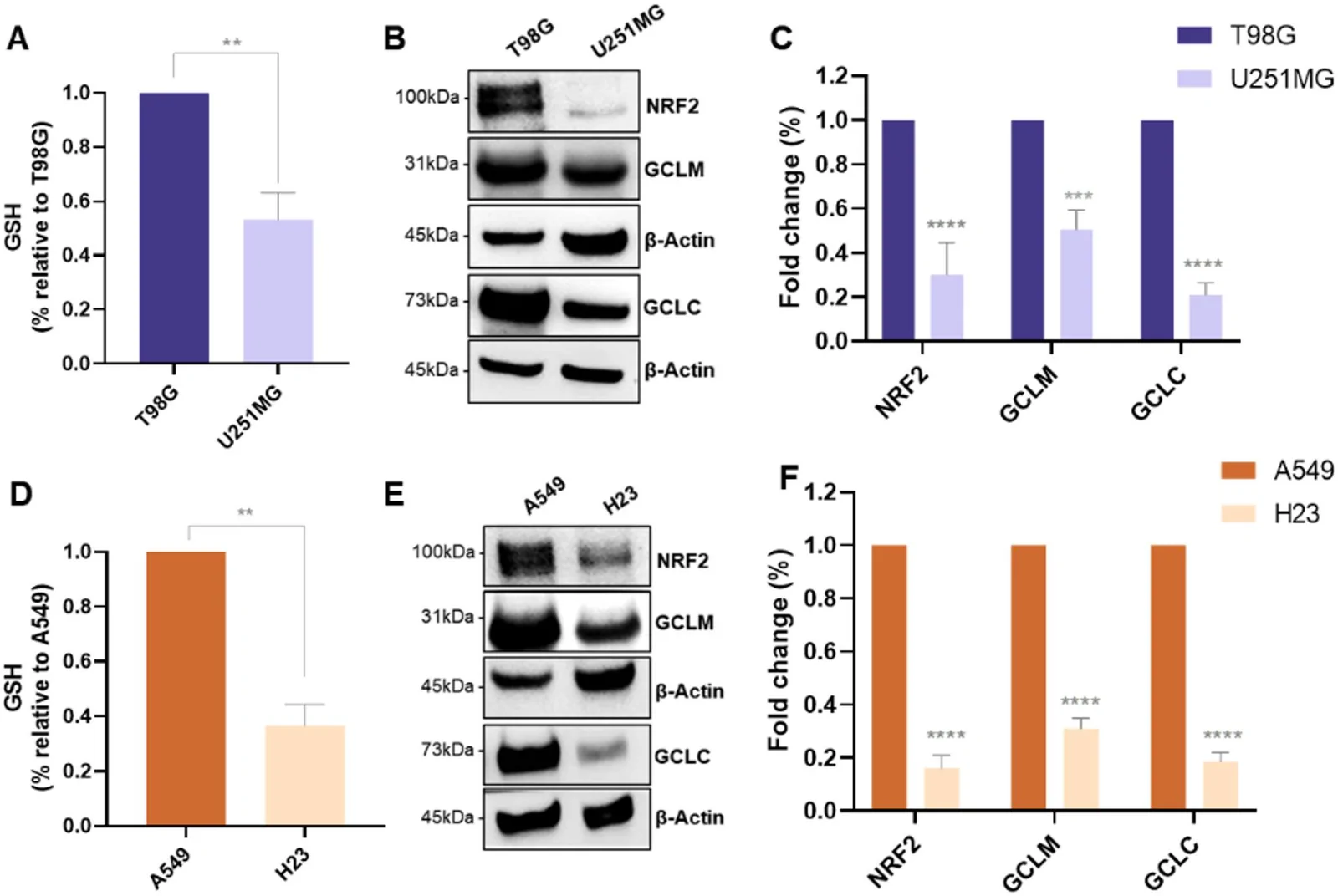
Expression of NRF2 targets and GSH availability. **A**, **D** Quantification of basal intracellular reduced GSH. **B**, **E** Comparison of NRF2, GCLM and GCLC protein expression by western blot. **C**, **F** Quantification of NRF2, GCLM and GCLC protein levels in glioblastoma and NSCLC cells. Values are mean ± SEM of three independent experiments, ns = not statistically significant, *P < 0.05, **P < 0.01, ***P < 0.001, ****P < 0.0001. Values were normalized to the respective control condition (1.0) for each independent experiment

Chemotherapy resistance is a complex phenomenon influenced by multiple factors [42]. One pivotal mechanism underlying resistance involves the activation of the antioxidant pathway, a process facilitated by the activity of glutathione (GSH) and the NRF2 signaling [43]. Therefore, we quantified the intracellular reduced GSH in our cell lines. Drug-sensitive cell lines exhibited lower levels of reduced GSH (Fig. 2A and D), as expected. Since NRF2 is the main precursor of GSH synthesis, by regulating GCLC and GCLM activity, we evaluated the expression levels of NRF2 and its target genes by western blot. Not surprisingly, NRF2, GCLC, and GCLM protein levels were significantly reduced in U251MG and NCI-H23 cells, compared with their respective chemotherapy-resistant control cells (Fig. 2B, C, E and F). These results indicate that high levels of NRF2, GCLC, and GSH may be related to chemotherapy resistance in glioma and lung cancer.

### NRF2 plays a key role in chemotherapy and BSO resistance

**Fig. 3:**
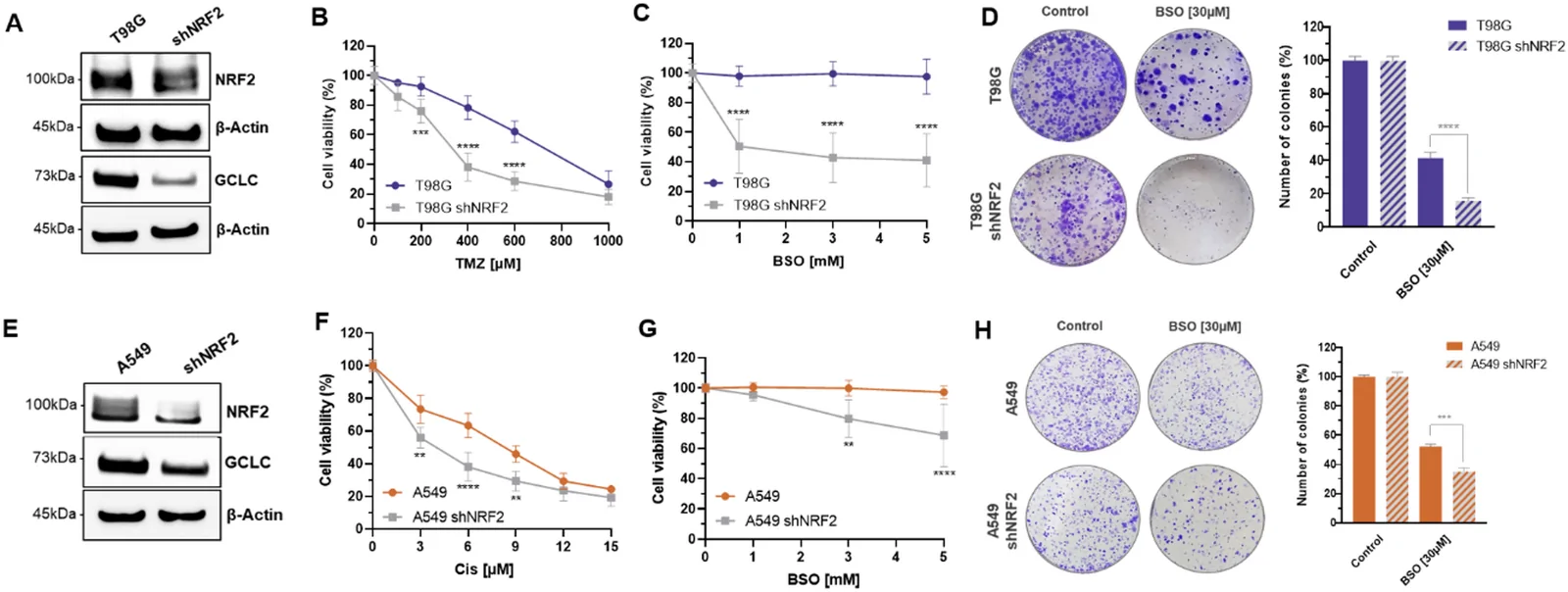
Cellular response of NRF2 silenced cells to chemotherapy and BSO. **A**, **E** NRF2 and GCLC detection protein by western blot of T98GWT, T98GshNRF2, A549WT and A549shNRF2. **B**, **C** Cell viability analyzed after treatment with TMZ (100 to 1000 µM) for 120 h or BSO (1 to 5 mM) for 72 h measured by XTT assay. Values are mean ± SD of three independent experiments. **D** Representative image and quantification of total colonies after BSO (30 µM) treatment in comparison to controls. **F**, **G** Cell viability analyzed after treatment with cisplatin (3 to 15 µM) or BSO (1 to 5 mM) for 72 h measured by XTT assay. **H** Representative image and quantification of total colonies after BSO (30 µM) treatment in comparison to controls. Values are mean ± SEM of three independent experiments, ns = not statistically significant, *P < 0.05, **P < 0.01, ***P < 0.001, ****P < 0.0001

To better understand the role of the signaling NRF2 pathway as a mediator of chemotherapy resistance, we silenced NRF2 in resistant cell lines (T98G and A549) using a shRNA lentiviral system. As shown in Fig. 3A and B, the silencing resulted in a substantial decrease in NRF2 and GCLC protein levels in both cell lines. Next, we evaluated the cell viability upon treatment with TMZ or cisplatin, and we observed that lower levels of NRF2 contributed to reducing the chemotherapy resistance in both cell lines (Fig. 3B and F). To further explore the relationship between NRF2 and GSH, the silenced cells were treated with BSO (buthionine sulfoximine) – a well-known inhibitor of y-glutamylcysteine synthetase (GCLC), which blocks GSH synthesis. Notably, T98GshNRF2 and A549shNRF2 cells were significantly more sensitive to BSO compared to control cells as shown by XTT cell viability assay (Fig. 3C and G). Similarly, NRF2 silencing significantly decreased the capacity of colony formation in T98G and A549 cells after BSO treatment (Fig. 3D and H). This data indicates that NRF2 contributes to TMZ resistance in glioblastoma and cisplatin resistance in NSCLC cells.

### NSCLC and glioblastoma cells with low levels of NRF2 are sensitive to BSO

**Fig. 4:**
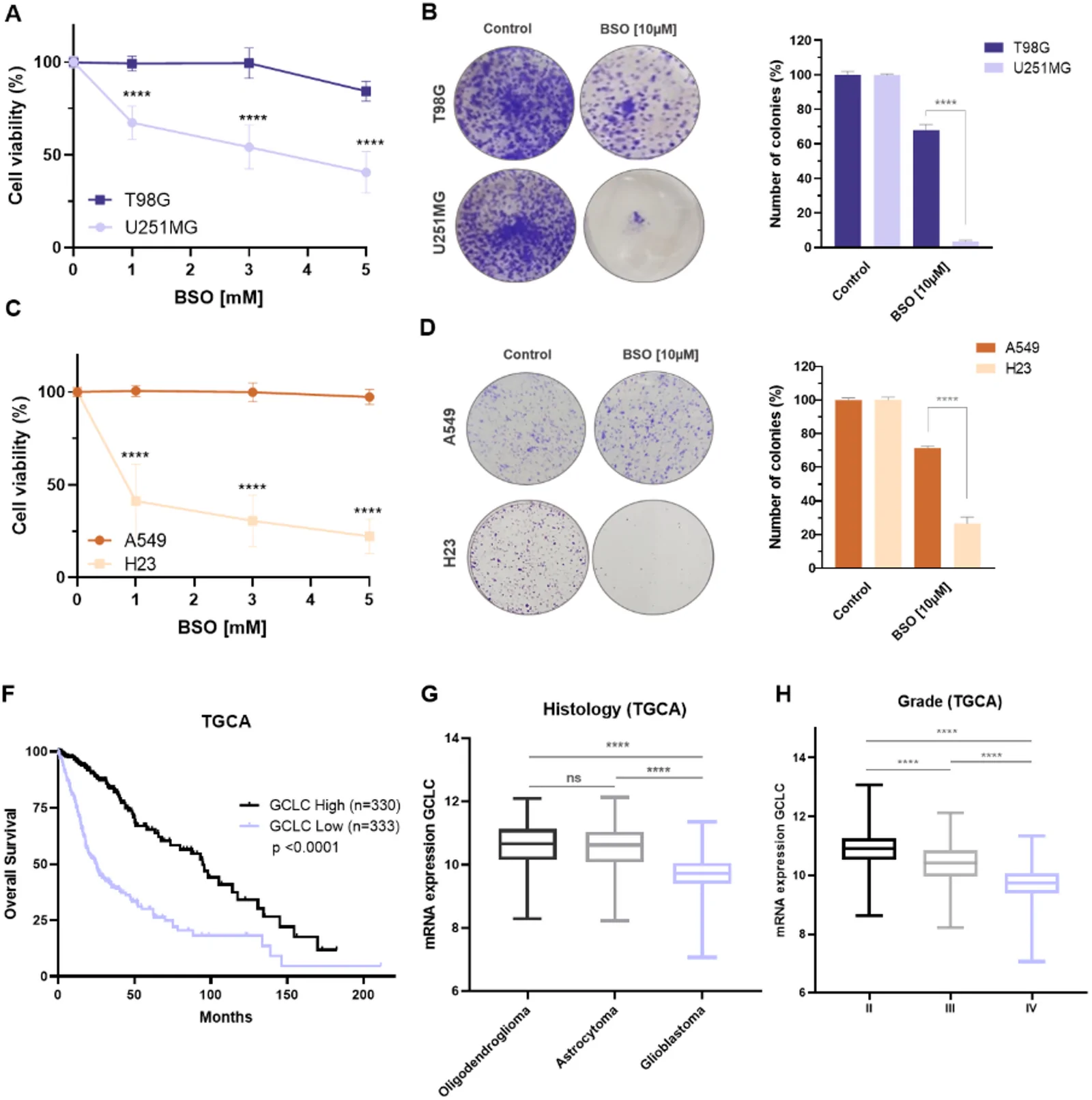
Differential sensitivity to BSO in glioma and lung cancer cells. **A**, **C** Cell viability analyzed after treatment with BSO (1 to 5 mM) for 72 h measured by XTT assay. Values are mean ± SD of three independent experiments. **B**, **D** Representative image and quantification of total colonies formed after BSO treatment (10 µM) in comparison to controls. **F** Kaplan–Meier curves showing overall survival of primary glioma patients from the TCGA cohort stratified according to GCLC expression. Patients were sub grouped into high GCLC expression (above median) and low GCLC expression (below median). **G** GCLC mRNA expression in the TCGA cohort of patients stratified by histology and **H** grade. Values are mean ± SEM of three independent experiments, ns = not statistically significant, *P < 0.05, **P < 0.01, ***P < 0.001, ****P < 0.0001

Since NRF2 silencing promoted sensitivity to BSO, we hypothesized that cells with low expression of NRF2 and GSH could be more responsive to BSO treatment. In fact, upon BSO treatment, U251MG and NCI-H23 were demonstrated to be highly sensitive, while T98G and A549 were marginally affected by treatment (Fig. 4A and C). Furthermore, BSO treatment impaired the colony formation in U251MG and NCI-H23 cells (Fig. 4B and D). To confirm that BSO reduces the level of GSH in these cells, we assessed the levels of reduced GSH after BSO treatment for 24 h and, as we can see in Supplementary Figure S1 online, BSO was able to greatly deplete the GSH intracellular levels in all cell lines. Since BSO-sensitive cells displayed lower levels of GCLC, we explored whether these gene expressions could be a potential biomarker for prognosis in glioblastoma or NSCLC. Interestingly, our analysis using the GlioVis data portal showed a shorter overall survival (OS) of glioma patients with lower levels of GCLC compared with patients with higher GCLC levels. Likewise, glioblastoma patients presented lower levels of GCLC in comparison to other glioma types (oligodendroglioma and astrocytoma), and high-grade glioma cases (according to WHO classification) displayed decreased levels of GCLC when compared to the other grades. Kaplan-Meier and mRNA expression analysis in CGGA and REMBRANDT databases confirmed low GCLC as a molecular marker of poorer overall survival in glioma (see Supplementary Fig. S2 online). Lung carcinoma patients showed no difference in overall survival when comparing patients with low and high GCLC. These data indicate that BSO therapy alone exhibits the potential to sensitize tumors characterized by low GCLC expression, indicating a rationale for the inclusion of BSO in the treatment regimen of this subgroup of patients.

### BSO sensitizes glioblastoma and NSCLC drug-resistant cells to chemotherapy

**Fig. 5:**
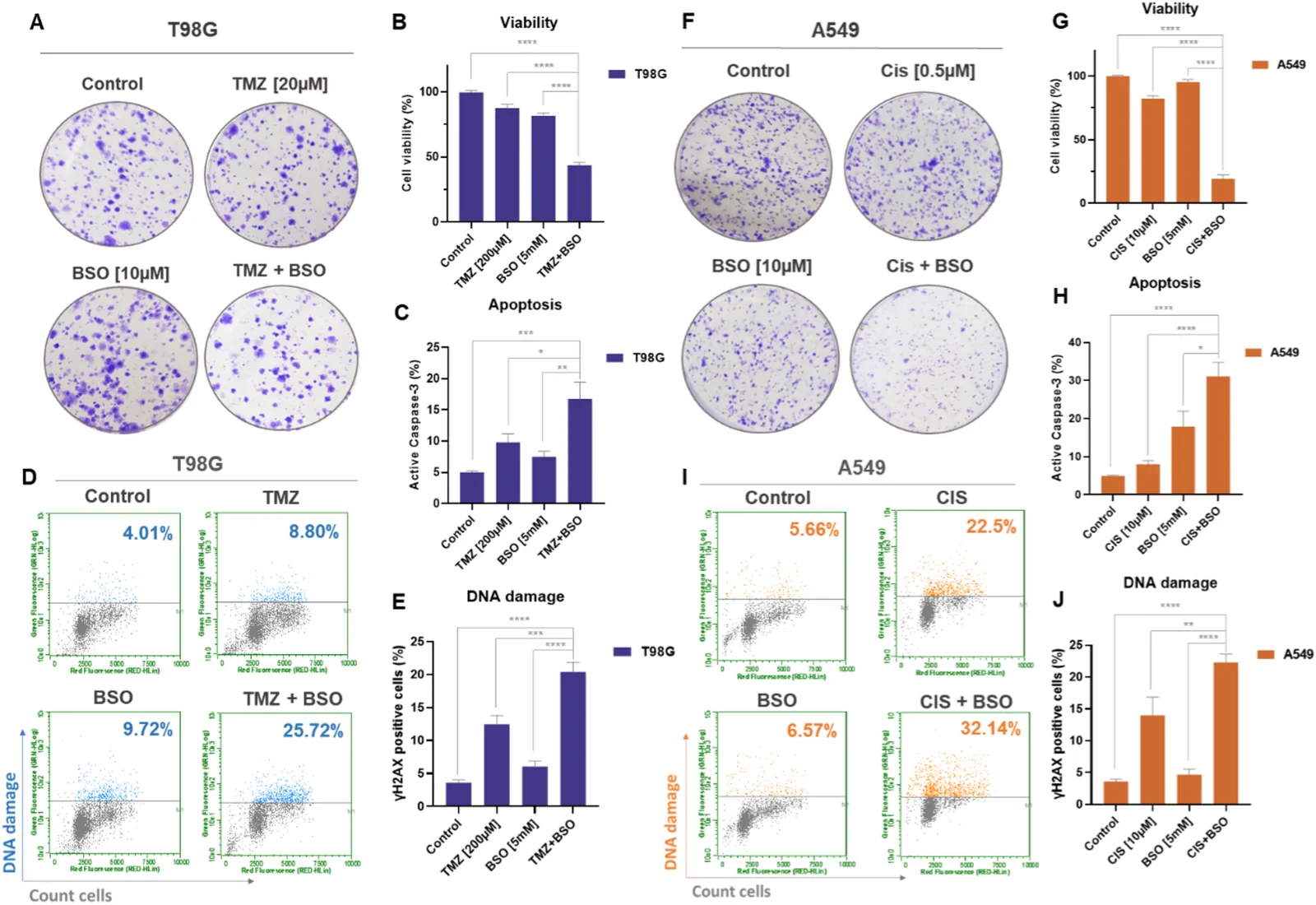
Cellular response after BSO and chemotherapy combinatorial treatment. **A** Representative image of total colonies after TMZ (20 µM) and BSO (10 µM) combinatorial treatment in comparison to controls in T98G cells. **B** Cell viability analyzed after combinatorial treatment with TMZ (200 µM) and BSO (5 mM) for 120 h measured by XTT assay in T98G cells. **C** Flow cytometry analysis of the percentage of active caspase-3 positive cells after combinatorial treatment with TMZ (200 µM) and BSO (5 mM) for 48 h in T98G cells. **D**, **E** Representative dot plot and quantification of the percentage of γH2AX positive cells after combinatorial treatment with TMZ (200 µM) and BSO (5 mM) for 48 h in T98G cells. **F** Representative image of total colonies after cisplatin (0.5 µM) and BSO (10 µM) combinatorial treatment in comparison to controls in A549 cells. **G** Cell viability analyzed after combinatorial treatment with cisplatin (10 µM) and BSO (5 mM) for 72 h measured by XTT assay in A549 cells. **H** Flow cytometry analysis of the percentage of active caspase-3 positive cells after combinatorial treatment cisplatin (10 µM) and BSO (5 mM) for 24 h in A549 cells. **I**, **J** Representative dot plot and quantification of the percentage of γH2AX positive cells after combinatorial treatment cisplatin (10 µM) and BSO (5 mM) for 24 h in A549 cells. Values are mean ± SEM of three independent experiments, ns = not statistically significant, *P < 0.05, **P < 0.01, ***P < 0.001, ****P < 0.0001

Since BSO was able to reduce GSH both in sensitive and resistant cell lines, we hypothesized that GSH modulation by BSO could enhance chemotherapy cytotoxicity in glioma and NSCLC-resistant cells. Thus, T98G and A549 were treated with TMZ and cisplatin, respectively, as single agents or in combination with a non-cytotoxic concentration of BSO. Clonogenic formation of these cells after combined treatment showed increased cell death compared to single treatment in both cell lines (Fig. 5A and F). Next, cell viability was measured by XTT assay, and we observed that cells co-treated with BSO and chemotherapeutic drugs presented a higher decrease in viability than those treated with TMZ or cisplatin alone (Fig. 5B and G). Levels of active caspase-3 were analyzed using flow cytometry and showed that the combined treatment increased apoptotic cell death in T98G and A549 (Fig. 5C and H). We also analyzed the effects of co-treatment in DNA damage induction by measuring γH2AX expression by flow cytometry. γH2AX was accumulated in both cell lines upon combinatorial treatment, indicating that BSO increases TMZ and cisplatin genotoxicity (Fig. 5D, E, I and J). Altogether, our results demonstrate that modulation of the NRF2/GSH-dependent antioxidant defense system by BSO enhances the sensitivity of drug-resistant NSCLC and glioblastoma cells to chemotherapy, likely through impaired redox adaptation and increased susceptibility to treatment-induced oxidative stress. These findings highlight the therapeutic potential of targeting the NRF2/GSH axis to overcome chemoresistance.

### Combinatorial treatment with BSO and cisplatin promotes apoptosis and ferroptosis in A549 cells

**Fig. 6:**
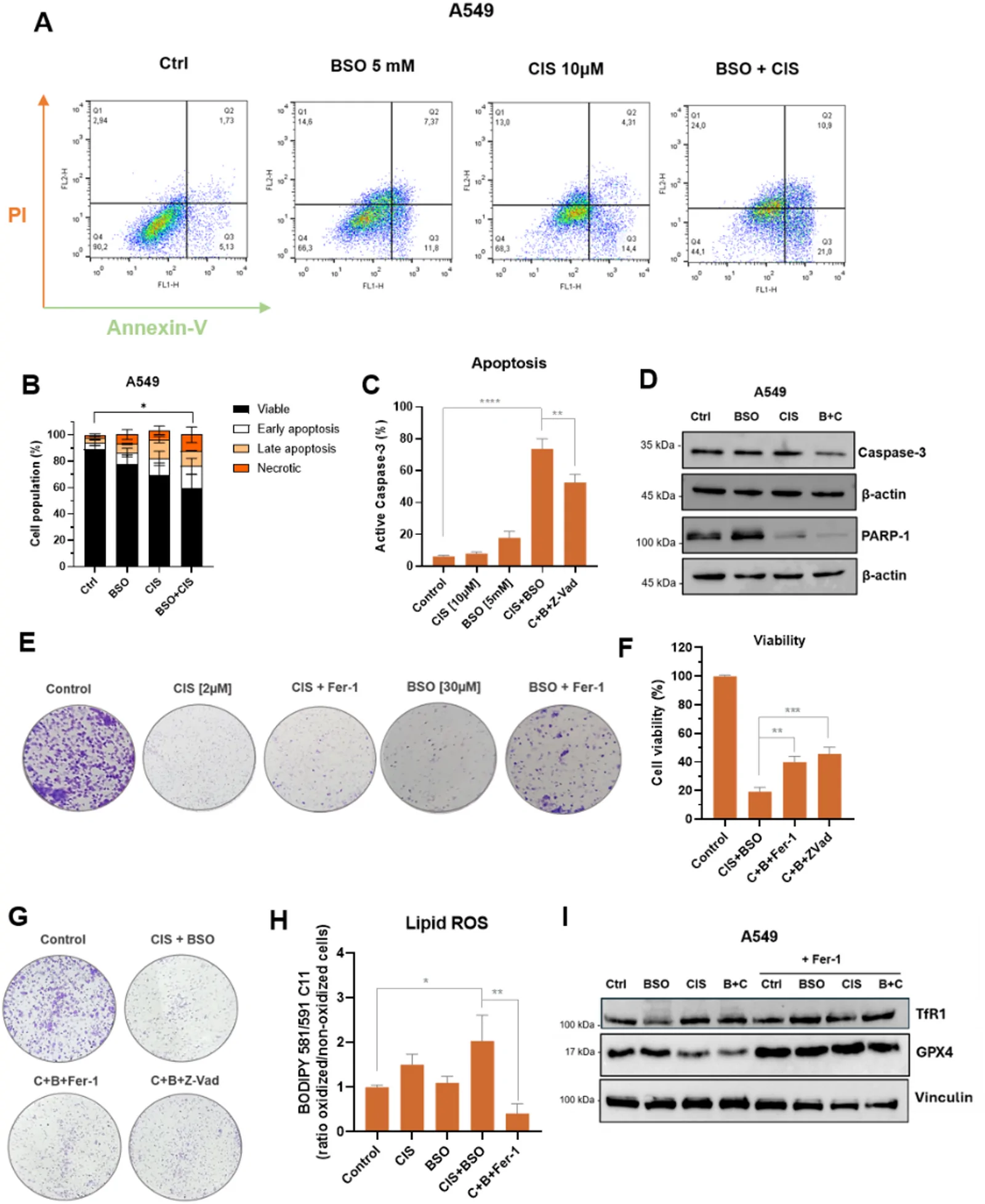
Cellular responses upon combinatorial treatment with BSO and cisplatin. **A** Representative Annexin V/PI flow cytometry plots of A549 cells following treatment with the indicated drugs alone or in combination. **B** Quantification of the percentage of cells in the viable, early apoptotic, late apoptotic, and necrotic populations. **C** Flow cytometry analysis of the percentage of active caspase-3 positive cells after treatment with cisplatin (10 µM), BSO (5 mM), and z-Vad (50 µM) for 24 h. **D** Representative Western blot analysis of total caspase-3, PARP-1, and β-actin protein levels in cells treated with the indicated combination. **E** Representative image of total colonies after cisplatin (2 µM), BSO (30 µM) and ferrostatin-1 (0.1 µM) treatment in comparison to controls. **F** Cell viability analyzed after treatment with cisplatin (10 µM), BSO (5 mM), ferrostatin-1 (0.1 µM) and z-Vad (50 µM) for 72 h measured by XTT assay. **G** Representative image of total colonies after cisplatin (0.5 µM), BSO (10 µM), ferrostatin-1 (0.1 µM) and z-Vad (5 µM) treatment in comparison to controls. **H** Lipid ROS was measured by the BODIPY-C11 probe (ratio oxidized/non-oxidized) after cisplatin (10 µM), BSO (5 mM), ferrostatin-1 (0.1 µM) treatment for 24 h. **I** Representative Western blot analysis of transferrin (TfR1) and GPX4 protein levels in A549 cells treated with the indicated drugs alone or in combination, in the presence or absence of ferrostatin-1 (Fer-1). Values are mean ± SEM of three independent experiments, ns = not statistically significant, *P < 0.05, **P < 0.01, ***P < 0.001, ****P < 0.0001

To analyze the mechanism of programmed cell death promoted by BSO and cisplatin alone or in combination, we first investigated whether the combinatorial treatment would promote ferroptotic and/or apoptotic cell death. For this first, using Annexin V/PI assay we observed that cisplatin and BSO co-treatment increased the proportion of cells in both early and late apoptosis, as well as the Annexin V/PI double-positive population, consistent with increased late-stage/secondary necrotic cell death (Fig. 6A, B). Consistent with these findings, the combinatorial treatment markedly increased the percentage of active Caspase-3-positive cells, which was partially rescued by Z-VAD-fmk (Fig. 6C). We further analyzed total Caspase-3 and PARP-1 protein levels by Western blot and observed a reduction in both proteins following combination treatment, providing complementary evidence of the apoptotic response (Fig. 6D).

We next investigated the ferroptosis pathway. First, we determined the clonogenic capacity of A549 cells after treatment with cisplatin or BSO alone or in combination with ferrostatin-1 (a specific inhibitor of ferroptosis). Our data demonstrate that the sensitivity to cisplatin or BSO was partially rescued by ferrostatin-1 treatment (Fig. 6E). Then, we investigated if the combinatorial treatment would promote ferroptotic or apoptotic cell death. We first evaluated cell viability following treatment with cisplatin and BSO in combination with ferrostatin-1 or Z-VAD-fmk, inhibitors of ferroptosis and apoptosis, respectively. Both inhibitors partially rescued cell viability (Fig. 6F) and modestly restored clonogenic capacity (Fig. 6G). To investigate whether the ferroptosis pathway is triggered by combinatorial treatment, we monitored the levels of lipidROS after treatment using the redox-sensitive dye BODIPY 581/591 C11. The co-treatment with cisplatin and BSO enhanced lipid peroxidation, which was rescued by ferrostatin-1 (Fig. 6H). Consistent with the induction of ferroptosis-related signaling, Western blot analysis revealed that the combination treatment reduced the levels of transferrin receptor (TfR1), an indicator of the iron-uptake component of ferroptotic signaling, and GPX4 in A549 cells, whereas co-treatment with ferrostatin-1 restored the levels of these proteins (Fig. 6I). Collectively, these findings indicate that the combined treatment of cisplatin and BSO promotes both apoptotic and ferroptosis-related responses in NSCLC cells.

In T98G cells, the combination treatment predominantly increased late apoptosis, and reduced total caspase-3 and PARP-1 protein levels, further supporting an apoptotic response (Fig. S3A, B,C). In parallel, Ferrostatin-1 failed to rescue the loss of viability and clonogenic survival induced by the combination and treatment alone (Fig. S3D, E,F). Curiously, combined treatment increased lipid peroxidation and altered ferroptosis-related protein levels (TfR1), changes that were restored by ferrostatin-1 (Fig. S3G, H). These findings indicate that the combination engages both apoptotic and ferroptosis-related responses; however, the ferroptotic response, although detectable, is not sufficient to account for the long-term cytotoxic effects in T98G cells.

## Discussion

In this study, we aimed to explore tumor heterogeneity and pursue novel alternatives to achieve more favorable outcomes for NSCLC and glioma patients. Thus, we conducted in vitro assays to investigate the differential sensitivity to chemotherapy of two cell lines from the same cancer subtype. We initially measured the cell viability after TMZ treatment in glioblastoma cells U251MG and T98G cells and after cisplatin treatment in lung cancer cells A549 and NCI-H23 cells. Our results revealed that U251MG and NCI-H23 are more sensitive to chemotherapy and present elevated levels of DNA damage after treatment, in contrast to T98G and A549.

Cisplatin is a DNA-reactive alkylating agent that forms covalent adducts with DNA, including monoadducts, as well as intra- and interstrand crosslinks. Among these lesions, interstrand crosslinks (ICLs) are regarded as the most cytotoxic, as they severely impair DNA replication and transcription preventing replication and transcription [8, 44]. Additionally, cisplatin has been observed to generate reactive oxygen species (ROS), necessitating a robust antioxidant defense system in tumor cells to evade cell death [45]. Similarly, TMZ induces cell death by DNA damage induction through DNA methylation at specific guanine and adenine positions [13, 18, 46]. The O6-methylguanine (O6-meG) lesion, the most toxic lesion induced by TMZ, can be repaired by O6-methylguanine methyltransferase (MGMT), and its expression correlates with tumor cell resistance [18]. Indeed, T98G bears an MGMT promoter unmethylated status, which greatly contributes to TMZ resistance [13, 18].

Like cisplatin, TMZ can induce ROS via mitochondrial DNA damage, implicating the antioxidant pathways as pivotal contributors to cisplatin and TMZ resistance [24, 25]. Notably, NRF2 has been implicated in chemotherapy resistance by regulating GSH synthesis in several types of cancer [41, 43, 48, 49]. Our previous investigations, involving U87MG, U343MG, U251MG, and U138MG cells, revealed that intracellular GSH concentration and NRF2 expression are critical for TMZ resistance in glioma [23, 26, 50]. Here, we extend these observations by demonstrating that T98G and A549 cells display elevated NRF2 activity, as evidenced by increased expression of its downstream targets GCLC and GCLM and the concomitant accumulation of intracellular glutathione. Notably, both pharmacological and genetic suppression of NRF2 significantly enhanced sensitivity to TMZ and cisplatin, highlighting the critical contribution of the NRF2/GSH antioxidant pathway to chemoresistance in these cancer models. Notably, this differential NRF2 activity is consistent with underlying genetic alterations, as T98G and A549 cells harbor NRF2 pathway-activating events (NRF2 amplification and loss-of-function KEAP1 mutation, respectively) [51, 52], whereas U251MG and NCI-H23 cells lack such alterations and exhibit lower NRF2 target gene expression and GSH levels.

Importantly, NRF2 silencing increased the sensitivity to GSH depletion by BSO, revealing a potential vulnerability in cancer cells [25]. BSO, a specific inhibitor of GCLC, blocks the rate-limiting step of GSH biosynthesis and therefore is a widely used agent for cellular glutathione depletion [34, 38]. Our hypothesis posits that cancer cells with basal low levels of GCLC would exhibit greater sensitivity to GSH inhibition than those with higher levels of GCLC and NRF2. Indeed, U251MG and NCI-H23 exhibited enhanced vulnerability to BSO compared to T98G and A549. Importantly, we showed that glioma patients with low levels of GCLC had a poorer prognosis than those with higher GCLC levels, emphasizing the need for innovative treatment modalities for this group of patients. In this context, the sensitivity to BSO treatment alone in tumors with lower levels of GCLC suggests a potential clinical rationale for the incorporation of BSO therapy into future therapeutic strategies for this subset of glioma patients.

Regarding drug-resistant cells, our investigation explored whether GSH depletion would increase the sensitivity of these cells to chemotherapy. Several studies have indicated that BSO can potentiate the toxicity of chemotherapy in various cancer types in vitro and in vivo [31, 35, 37, 39]. The results herein demonstrate that GSH-depleted cells by BSO exhibited significantly decreased viability after TMZ and cisplatin treatment, inducing higher levels of DNA damage and active caspase-3. This suggests that high levels of GSH contribute to reduce TMZ and cisplatin cytotoxicity within cells, corroborating prior research demonstrating that intracellular GSH is determinant to resistance to chemotherapy in glioma and lung cancer cells [23, 26]. More importantly, the combined treatment with BSO could potentiate current chemotherapeutic treatments. BSO combined with TMZ or cisplatin displayed a synergistic effect on NRF2-overexpressing cells. Crucially, NRF2 mutations or loss-of-function mutations of KEAP1 are the most prevalent type of mutation in NSCLC tumors, leading to increased expression of NRF2 and therefore poorer clinical outcomes [53, 54]. In such cases, BSO combined therapy may represent a potential strategy to sensitize NRF2-high tumors; however, its therapeutic efficacy and safety require validation in preclinical and clinical models.

Several research groups have undertaken phase I clinical studies to determine whether BSO achieves the desired biochemical endpoint of GSH depletion in vivo [55, 56]. Preliminary studies revealed that continuous infusion of BSO was partially non-toxic and led to tumor GSH depletion in patients with advanced cancers [33, 57, 58] Our results provide preclinical evidence supporting further investigation of BSO alone in drug-sensitive tumors and as an adjuvant to chemotherapy in resistant tumors. However, the clinical impact of BSO administration requires further investigation *in vivo.*

GSH activity is recognized as crucial for protection against a recently described type of cell death called ferroptosis, therefore BSO has been extensively related to ferroptosis induction [38, 59, 60]. Our findings demonstrate that cisplatin alone exerts a weak ferroptosis-related response in A549 cells. In contrast, BSO-induced cell death was rescued by ferroptosis inhibitor. Interestingly, the combinatorial treatment of cisplatin and BSO was able to trigger both apoptotic and ferroptosis-related cell death in NSCLC. In this study, we showed that cisplatin and BSO combined treatment can engage ferroptotic signaling, as evidenced by increased lipid peroxides levels within the cells and changes in ferroptosis-related proteins that were reversed by ferrostatin-1. This discovery suggests that targeting GSH metabolism may promote ferroptosis-related responses in chemotherapy-resistant cells and warrants further investigation.

In addition to ferroptosis-related responses, the combination induced a marked apoptotic response. Annexin V/PI analysis revealed increased early and late apoptotic populations in A549 cells, together with an increase in Annexin V/PI double-positive cells, whereas T98G cells predominantly displayed an increase in late apoptosis. Consistently, combination treatment increased active caspase-3 levels and reduced total caspase-3 and PARP-1 protein levels in both cell models, further supporting the involvement of apoptosis in the response to combined treatment. Together, these findings indicate that BSO and cisplatin engage multiple cell death-associated pathways, which holds particular significance for patients who have developed resistance to treatments that induce only apoptosis.

Several limitations of this study should be considered. First, our conclusions are based primarily on in vitro experiments using a limited number of established cancer cell lines, which may not fully recapitulate the genetic, metabolic, and microenvironmental heterogeneity of human tumors. Second, although our data provide evidence for the involvement of both apoptotic and ferroptosis-related responses, additional mechanistic studies and complementary models will be required to define the relative contribution of each pathway. In particular, the absence of in vivo models represents an important limitation, as the therapeutic efficacy, pharmacokinetics, toxicity, and tumor selectivity of BSO in combination with chemotherapy cannot be determined from the present experiments. Future studies using xenograft or genetically relevant in vivo models, as well as additional patient-derived or genetically diverse models, will be important to validate these findings and establish their translational relevance.

### Conclusion

Overall, our findings highlight GSH metabolism and NRF2 activity as important determinants of chemotherapy response across distinct tumor models and provide evidence that pharmacological GSH depletion with BSO can enhance the response of NRF2-high, chemotherapy-resistant cells to TMZ or cisplatin. The engagement of both apoptotic and ferroptosis-related responses following combination treatment further underscores the potential of targeting cellular redox homeostasis to overcome chemotherapy resistance. However, validation in more physiologically relevant models and in vivo systems will be essential to establish the translational potential of this strategy.

## Supplementary Information

Below is the link to the electronic supplementary material.

Supplementary material 1 (DOCX 763.6 kb)

Supplementary material 2 (DOCX 4330.9 kb)

## Data Availability

The datasets generated and/or analyzed during the current study are available from the corresponding author on reasonable request.
