# Supplementary material for: NRF2 signaling drives chemoresistance in NSCLC and glioblastoma
Source: Mol Cell Biochem. 2026 Sep 19;481(10):5577–90. doi: 10.1007/s11010-026-05747-z (PMC13687227; doi:10.1007/s11010-026-05747-z)
Supplement: Supplementary file 1 [file 11010_2026_5747_MOESM1_ESM.docx]

**Supplementary Figure 1**

**Figure S1. Analyse of GSH levels. (A,B)** Quantification of intracellular reduced GSH after treatment with BSO (5 mM) for 24 h. Values are mean ± SEM of three independent experiments, ns = not statistically significant, *P < 0.05, **P < 0.01, ***P < 0.001, ****P < 0.0001. Data were normalized to the respective control condition, which was set to 100% in each independent experiment. Values were normalized to the respective control condition (1.0) for each independent experiment.

**Supplementary Figure 2**

**Figure S2. GCLC expression analysis of glioma patients. (A,D)** Kaplan–Meier curves showing overall survival of glioma patients from the CGGA and REMBRANDT cohort stratified according to GCLC expression. Patients were subgrouped into high *GCLC* expression (above median) and low *GCLC* expression (below median). **(B,E)** GCLC mRNA expression in the CGGA and REMBRANDT cohort of patients stratified by histology; **(C,F)** GCLC mRNA expression in the CGGA and REMBRANDT cohort of patients stratified by grade. ns = not statistically significant, **P* < 0.05, ***P* < 0.01, ****P* < 0.001, *****P* < 0.0001.

**Supplementary Figure 3**

**Figure S3. Cellular responses upon combinatorial treatment with BSO and TMZ in T98G cells.** (A) Representative Annexin V/PI flow cytometry plots of T98G cells following treatment with the indicated drugs alone or in combination. (B) Quantification of the percentage of cells in the viable, early apoptotic, late apoptotic, and necrotic populations. (C) Representative Western blot analysis of total caspase-3, PARP-1, and β-actin protein levels in cells treated with the indicated combination. (D) Representative image of total colonies following treatment with TMZ (50 µM), BSO (30 µM) and ferrostatin-1 (0.1 µM) compared to control conditions. (E) Cell viability following treatment with BSO (5 mM) and ferrostatin-1 for 72 h, measured by XTT assay. (F) Cell viability following treatment with TMZ (200 μM) and BSO (5 mM) and ferrostatin-1 for 120 h, measured by XTT assay. (G) Lipid peroxidation was assessed by BODIPY-C11 fluorescence (oxidized/non-oxidized ratio) after treatment with TMZ (200 μM) and BSO (5 mM) for 24 h. (H) Representative Western blot analysis of transferrin (TfR1), and vinculin protein levels in T98G cells treated with the indicated drugs alone or in combination, in the presence or absence of ferrostatin-1 (Fer-1). Values are presented as mean ± SEM from three independent experiments. Data were normalized to the respective control condition, which was set to 100% in each independent experiment. Statistical significance was determined as indicated: ns, not statistically significant; *P < 0.05; **P < 0.01; ***P < 0.001; ****P < 0.0001.
