# Supplementary material for: NRF2 signaling drives chemoresistance in NSCLC and glioblastoma
Source: Mol Cell Biochem. 2026 Sep 19;481(10):5577–90. doi: 10.1007/s11010-026-05747-z (PMC13687227; doi:10.1007/s11010-026-05747-z)
Supplement: Supplementary file 2 [file 11010_2026_5747_MOESM2_ESM.docx]

**Uncropped western blot images**

After blockage, the membranes were cropped according to the molecular weight of the proteins to be detected.

**Figure 2B**

**Figure 2E**

**Figure 3A**

*The ladder image was not detected in the right samples.

**Figure 3E**

*The ladder image was not detected in this blot.

**Figure 6D**

**Figure 6I**

**Figure S3C**

**Figure S3H**

*****Red boxes indicate the cropped areas that were used in the figure. Western blot membranes were sequentially stripped and reprobed as indicated.
